# Occupant behaviour as a fourth driver of fuel poverty (aka warmth & energy deprivation)

**DOI:** 10.1016/j.enpol.2019.03.023

**Published:** 2019-06

**Authors:** Ade Kearns, Elise Whitley, Angela Curl

**Affiliations:** a Urban Studies, School of Social and Political Sciences, University of Glasgow, 25 Bute Gardens, Glasgow, G12 0NU, UK; b MRC/CSO Social and Public Health Sciences Unit, 200 Renfield Street, Glasgow, G2 3QB, UK; c Department of Population Health, University of Otago, Christchurch, PO Box 4345, Christchurch, New Zealand

**Keywords:** Fuel poverty, Occupant behaviour, Affordability, Vulnerability, Longitudinal

## Abstract

A conceptual framework for occupant behaviour as a driver of fuel poverty is presented, comprising: housing and use of the home; heating and energy arrangements and thermal comfort; household structure and dynamics; health and well-being; household finances; and social activity and relations. This framework informs longitudinal analysis of movements into and out of fuel poverty among households in deprived communities in Glasgow. Household surveys across ten years yielded a longitudinal sample of 3297 cases where initial and subsequent fuel poverty status was recorded using an experiential measure. A third of households changed their fuel poverty status over time: 18% moving out of fuel poverty and 16% moving in. Factors strongly associated with movements into fuel poverty included: being a single parent (OR 2.27); experiencing a mental health problem (OR 2.74); and remaining out of work (OR 1.89). Movement out of fuel poverty was less likely among those with infrequent family contact (OR 0.55) and who moved home (OR 0.66); home improvements had no effect upon the experience of fuel poverty. It is argued that the policy problem should be considered one of ‘warmth and energy deprivation’, accompanied by a broader interpretation of vulnerability *to* as well as *from* fuel poverty.

## Highlights

• Fuel payment difficulties are lower than official fuel poverty rates.
• People with mental health problems are more likely to enter fuel poverty.
• Family support networks are protective against fuel poverty.
• Higher standards of energy efficiency may be required from interventions.
• Wider efforts are needed to identify those at risk of warmth and energy deprivation.

## 1 Introduction

Despite policy aims to eradicate fuel poverty, it remains an enduring policy problem in the UK, with higher rates in UK peripheral regions such as Scotland.^1^ (NEA, 2018). As a result UK governments have sought to redefine fuel poverty, with a new measurement in England (Hills, 2012) and an alternative proposed for Scotland (Scottish Government, 2017a). Definitions generally comprise elements that are easily measured, modelled and monitored so that policy progress can be estimated. They also reflect policy preferences to focus on things readily impacted through regulatory and direct policy instruments (Knoepfel et al., 2007). The prevailing view is that fuel poverty is driven by incomes, energy prices, and home energy efficiency.

Attention to the role of individuals in fuel poverty does not go far enough. Interest in mental health focuses on how cold homes impact mental health, rather than how mental health might affect fuel poverty (Liddell and Guiney, 2015). Occupant behaviour, an explanatory factor for urban-rural differences in fuel poverty, is restricted to the (in)ability to use newly installed heating systems (Mould and Baker, 2017a). Scope for considering individual characteristics and behaviours is expanded by the focus on household vulnerability, both in the definition of fuel poverty (Bramley et al., 2017) and the regulation of how energy companies treat their customers (Bartl, 2010). However, this focuses on people deemed vulnerable to the consequences of cold homes, such as the elderly and disabled, rather than considering how vulnerability may predispose people to fuel poverty.

This paper contributes to fuel poverty research and debate in three areas. Conceptually, it sets out a view of occupant behaviour as an additional driver of fuel poverty, broadening this beyond the use of heating controls and acquisition of thermal comfort after home improvements (Thomson et al., 2017). Additionally, an alternative understanding of the problem is proposed, not as ‘fuel poverty’ but as ‘warmth and energy deprivation’, which would allow consideration of issues of capability as well as affordability. Empirically, the paper adds valuable new evidence through the analysis of longitudinal rather than cross-sectional data, the use of a subjective or ‘consensual measure’ of fuel poverty (Thomson et al., 2017), and the incorporation of a wider range of occupant-related variables in the analysis (Huebner et al., 2015). The paper examines new questions including: whether housing improvements are effective in reducing the ‘lived experience’ of fuel poverty (McIntosh and Wright, 2018); how movements into and out of fuel poverty compare; what household, housing and social factors are associated with these fuel poverty dynamics, and whether those factors are the same in each direction. The paper contributes to policy debate by opening up discussion about: what aspects of occupant behaviour might be acted upon if traditional housing interventions are ineffective; the value of adopting more than one type of measure of fuel poverty; and the need to expand the notion of vulnerability as currently contained within UK policy.

## 2 Understanding fuel poverty and the role of occupant behaviour

### 2.1 The official approach to understanding and defining fuel poverty

The way fuel poverty is defined in policy terms highlights the reliance upon three main drivers and the preference for conceptual simplicity, limiting the scope for considering behavioural factors. UK jurisdictions have traditionally relied upon measures of fuel poverty based on relative expenditure. For many years this was based on Boardman's 10% threshold (Boardman, 1991), to identify households spending an above average proportion of income on heating (Boardman, 2012) or fuel (Isherwood and Hancock, 1979), or needing to spend more than 10% of its income on heating and fuel (DEFRA, 2001; Scottish Executive, 2002). Whilst the 10% indicator is flexible, easy to calculate and easily understood, it has come to be seen as imprecise and unresponsive. Rather than identifying the ‘unusual or abnormal’ (Liddell et al., 2012, p.28), it was thought to bring too many people into the ambit of fuel poverty (Heindl, 2015), and to include many higher income households living in energy inefficient homes (Romero et al., 2018). The indicator did not help to target assistance to those in most need, nor demonstrate the effectiveness of energy efficiency measures. This was a particular problem in the colder regions of Northern Ireland and Scotland, where the larger numbers in fuel poverty were not dropping despite expenditures on energy efficiency programmes, and where fuel costs were higher than in other parts of the UK (Liddell et al., 2011).

UK jurisdictions now differ in their approach to fuel poverty. England has adopted the Low Income High Cost (LIHC) indicator proposed by Hills (2012), whereby a household is in fuel poverty if its required equalized fuel costs are above the national median level for all households, and its residual income after fuel costs is less than the official poverty line (less than 60% of equalized median income). However, LIHC has been criticised for not taking household and dwelling size into account, thereby omitting an important fuel poor group on low incomes in small dwellings (Moore et al., 2012; Walker et al., 2014), and for being insensitive fuel price changes (Bramley et al., 2017). Scotland has adopted a Minimum Income Standard (MIS) measure of fuel poverty that claims to be accurate, consistent (Moore, 2012) and capable of adjustment by type of household and region (Romero et al., 2018), having been applied to Italy (Valbonesi et al., 2014) and Germany (Heindl, 2015). The MIS is based on costing agreed elements of a decent standard of living for different kinds of households (Hirsch et al., 2016). Thus, a household is in fuel poverty if it needs to spend more than 10% of its income (after housing costs) on heating and electricity, and thereafter it has less than 90% of the national MIS as its residual income (Scottish Government, 2017a).

These approaches to defining fuel poverty enable national governments to monitor progress of programmes but have less utility at a local level, or for targeting households in need of assistance (Liddell et al., 2012). They are founded on a view that there are three drivers which can be technically measured or modelled and addressed by policy: energy efficiency, energy prices and incomes (Baynham-Hughes, 2013; Scottish Government, 2012). However, official approaches to fuel poverty substitute required expenditures for actual expenditures (based on occupant behaviours), with modelling assumptions criticised for being ‘insensitive to cultural differences in the use of rooms’ (Thomson et al., 2017, p.6; Todd and Steele, 2006). Moreover, the focus on the three drivers lacks consideration of household socio-demographic circumstances and energy needs (Thomson et al., 2017; Bouzarovski et al., 2012).

### 2.2 Consideration of behavioural factors

We clarify the need for a broader perspective, reviewing how occupant behaviour has featured in official approaches to defining fuel poverty. One way is in respect of under-occupancy, where, in England, the required heating regime is reduced. In Scotland under-occupancy is not adjusted for, since under-heating or zonal heating of the home would lead to damp, mould and structural problems (Scottish Government, 2012) and can be detrimental to health (Bramley et al., 2017). However, zonal heating can reduce estimates of fuel poverty and benefit households who are out during the day (Beizaee et al., 2015). Moreover, the description of the relationship between under-occupancy and combined fuel-and-income-poverty as ‘relatively uncommon’ seems misplaced given that over half the latter group under-occupy their homes in Scotland (Bramley et al., 2017p.71, table 4.8).

Occupant behaviour has usually been considered in terms of its influence on the effectiveness of interventions, rather than the occurrence of fuel poverty. In terms of exercising control, the Warm Front programme in England has reported contrasting effects.^2^ Mental health gains for occupants included reduction in anxiety and worry accompanying ‘having a reliable, controllable source of heat and hot water’ (Gilbertson et al., 2006, p.952). However, a quarter of recipients continued to live in cold homes, due to difficulties with ‘complicated’ programmers (Critchley et al., 2007, p.154), and slow adjustment to the possibility of living in warmer homes (Critchley et al., 2007). Social landlords in Scotland also report that, following improvements, occupants need advice and support to use new technologies, such as solar panels, and also more conventional heating systems (Changeworks, 2014; Faulk, 2015).

Most attention to occupant behaviour relates to the rebound effect following energy efficiency interventions. Households may decide to ‘take-back’ some of the gain by increasing the use of heating to raise their thermal comfort (Berkhout et al., 2000; Greening et al., 2000), perhaps also increasing their physical comfort by wearing less clothing, a so-called ‘take-off’ effect (Hong et al., 2009). The rebound effect has been studied in Europe, USA and China, with estimates of the lost energy savings ranging from 30% to 74% (e.g. BelaΪd et al., 2018; Haas and Biermayr, 2000; Thomas and Azevedo, 2013; Wang et al., 2014). There have been calls for better data collection on household lifestyles, energy use, and conservation behaviours to better predict energy savings (BelaΪd and Garcia, 2016; Sardianou, 2007) and it is recognised that attitudes matter as well as behaviours (BelaΪd et al., 2018).

### 2.3 Health-related vulnerability

Vulnerability is taken into account in a limited way in current policy in order to aid targeting (DECC, 2015), focusing on posterior effects rather than anterior susceptibility. Consequently, ‘it is likely that households with vulnerable members … will fall through the cracks’ (Middlemiss, 2017a, p.434). Policy identifies as vulnerable those people whose physical health is most at risk from living in cold homes. In England this includes older people, the long-term sick or disabled, and families with young children (DECC, 2015); in Scotland it has included people over 60 or disabled, but not families (Matthews, 2014). In both countries, ‘health-related vulnerability’ is seen as a behavioural issue with the vulnerable consuming more energy due to keeping their home warmer for longer, having additional hot water needs, and using electrical equipment to support independent living. Therefore, internal temperatures used for modelling energy requirements in defining fuel poverty are increased for some groups. However, a Scottish recommendation that minimum residual income be increased for households with a disabled member was rejected (Scottish Government, 2017a).

This narrow view of the relevance of occupant behaviour in identifying fuel poverty, confined to energy consumption patterns as affected by health-related vulnerability, is partly due to the reliance on a limited number of metrics in the estimation of the prevalence of fuel poverty and the ‘inclination to analyse problems technically rather than socially’ (Middlemiss, 2017a, p.439). Fuel poverty is a complex behavioural issue, and ‘people react in different ways to it (e.g. by not heating, going into debt, trading off other costs)’ (Middlemiss, 2017b, p.3). As a result, measures of fuel poverty based on expenditure or temperature data alone can present an inaccurate picture due to their failure to take into account everyday household practices and shifting expenditure priorities (Thomson et al., 2017, p.20).

### 2.4 A broader understanding of occupant behaviour

The use of subjective or ‘consensual indicators’ of fuel poverty, or in combination with objective measures (Thomson et al., 2016, 2017), introduces the possibility of considering occupant behaviour as a driver of fuel poverty, not only in terms of ‘how energy is used in the home’ due to physical health needs or after energy efficiency improvements (Sigsworth, 2016). For this purpose, a broader understanding of human behaviour can be helpful.

**Fig. 1:**
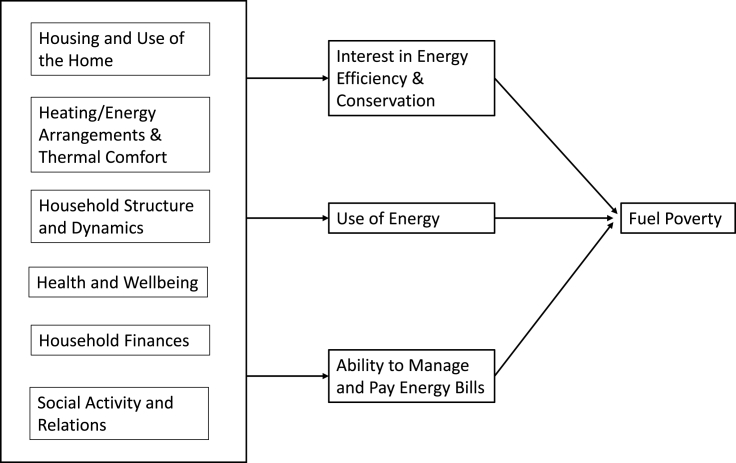
Conceptual model of occupant behaviour as an influence upon fuel poverty.

Human behaviour is defined as how people respond to internal and external stimuli, including both physical actions and observable emotions. Behaviour is influenced by thoughts, feelings, attitudes, values, social interaction and culture (Wikipedia, 2018). The context for human behaviour includes physical conditions, emotional state, cognitive capabilities and social status. Behaviours can be instinctive, learned, deliberative, or driven by physical needs or emotions (Schmidt, 2000). Socially, behaviour comprises four main phenotypes: envious, optimist, pessimist and trustful (Poncela-Casasnovas et al., 2016). On this basis, a broader interpretation of the influence of occupant behaviour on fuel poverty can be developed (Fig. 1). Six domains (housing and use of the home, heating arrangements, household structure and dynamics, health and wellbeing, finances, and social activity & relations) are viewed as reflecting occupant behaviour and contributing to fuel poverty through their influence upon the use of energy, attitudes towards energy efficiency and conservation, and ability to manage and pay energy bills. The UK government has concluded that the impacts of climate change mitigation measures on household energy bills depends partly upon occupant behaviour in taking up insulation measures and replacing energy-intensive products (DECC, 2013). The six domains are described below and their relevance illustrated through existing research on energy use and fuel poverty.

In relation to *housing and use of the home*, issues of choice are important. Firstly, dwelling type affects energy demand (Hamilton et al., 2013), with flats often more energy efficient than houses (Scottish Government, 2016a) and considered a reflection of housing choice or behaviour (Huebner et al., 2015). Second, housing tenure has effects as tenants may have less control over their energy supply and the type and operation of their heating system. How people use space within their homes can also affect their energy and heating demands, in terms of under- or over-occupation and which rooms household members regularly use. Energy consumption is affected by the number of electrical appliances, whether they are left on continuously or on stand-by (Firth et al., 2008), and whether occupants are concerned about energy saving and aware of their appliances' energy ratings (Yohanis, 2012).

*Heating/energy arrangements and thermal comfort* reflects several aspects of occupant behaviour. First, choice of energy supplier and willingness to switch: low income consumers are much less likely to switch supplier than higher income consumers (Hogan, 2016). Second, ‘many users do not use their heating controls effectively or at all’ (Consumer Focus, 2012, p.4) due to heating system complexity (Rathouse and Young, 2004) and occupants' motives to be ‘active’ or ‘passive’ users of their heating (EST and DCLG, 2010; Fabi et al., 2013). Third, perceptions of thermal comfort, psychological adaptation to temperatures based on personality, habituation and expectations, and attitudes to energy conservation influence use of heating (Dear de and Brager, 1998). Fuel poverty is over-estimated where ‘adaptive thermal comfort’ (using windows for ventilation, changing clothing, psychological tolerance of different temperatures) is ignored (Perez-Fargallo et al., 2017). Similarly, although in France a lower rate of fuel poverty is reported for those with electrical heating, such households may struggle to achieve thermal comfort (BelaΪd, 2018).

*Household structure and dynamics,* and *health and wellbeing,* are sometimes considered as socio-demographic covariates. Although they reflect aspects of occupant behaviour. Household structure is often considered in assessments of fuel poverty, with larger and foreign families more likely to be in fuel poverty (BelaΪd, 2018). *Household dynamics* covers changing household membership and relationships, affecting the use of energy and payment of bills, for example, the presence of children influencing heating zones and times (Love, 2014; de Chavez de et al., 2017). Instability in family life is very common among low-income households and causes material instability due to low incomes and lack of savings (Hill et al., 2017).

*Health and wellbeing* covers non-communicable diseases (NCDs), health behaviours and mental health, with problems more common in low income communities (Stafford et al., 2018). NCDs (e.g. cardiovascular diseases, cancers, chronic respiratory diseases and diabetes) and associated unhealthy behaviours (smoking, salt intake, alcohol use and low physical activity) are closely related to poor mental health (WHO, 2017). Vulnerability therefore includes occupant behaviour influenced by poor mental health, which may cause people to restrict their use of energy and have a poor heating regime (Mould and Baker, 2017a) or may result in people being unable to organise their household finances and manage their debts, leading to sustained fuel poverty with or without adequate warmth. Mould and Baker's interpretation of vulnerability can be extended to: “Anything which challenges a household's ability to keep warm/cool, *or to manage and pay their energy bills*”, recognising that people in similar housing circumstances may experience those conditions and be vulnerable in different ways (Middlemiss and Gillard, 2015).

*Household finances* covers equalized incomes, but also the level and duration of experience of low income and debt, and stability of income, all of which affect a household's ability to pay their energy bills. Householders differ in how they pay for energy, with low income households more likely to pay higher proportionate energy costs via pre-payment meters (Hogan, 2016). Households also vary in their response to constrained household budgets: some limit their use of heating to save money while others reduce other items of expenditure, such as food, to pay for heating (Grey et al., 2017; Harrington et al., 2005). In some cases, fear of being cut-off leads to prioritisation of fuel over food (Trevisan et al., 2014). The importance of budgeting is demonstrated by high variation in the absolute and relative amounts of income spent on fuel by low income households (Mould and Baker, 2017b; Middlemiss and Gillard, 2015).

The last domain covers *social activity and relations* outside the home. Most often, social relations are seen as a victim of fuel poverty, with households refraining from inviting others to their cold homes (Grey et al., 2017). But good social relations are also a potential protector from fuel poverty. External relations with wider family members, friends and neighbours are an important source of practical, emotional and financial advice and support. These may help people cope with fuel poverty and influence behaviours such as heating regimes, money management, and use of the home. The use of public spaces and amenities such as libraries as social spaces (Herrera-Viedman and Lopez-Gijon, 2013), also influences domestic energy consumption and susceptibility to fuel poverty. Social engagement around climate change, awareness of social norms, and information on environmental and climate change impacts of energy saving are partly a product of social activity and influential upon energy conservation (Lorenzi et al., 2007; Abrahamse and Shwom, 2018; Abrahamse et al., 2007).

Previous work in this area has been largely based on cross-sectional analyses, making it difficult to establish the direction of any associations. In addition, assessment of fuel poverty is generally defined in terms of UK jurisdiction on the percentage of household income spent on fuel, or a variation thereof. This type of measure does not capture the complexities of household budgeting and the more subjective, lived experience of fuel poverty. Finally, investigation is generally limited to those behaviours related to fuel use, most commonly following energy efficient home improvements. The role of other types of behaviours in determining whether a household enters or leaves fuel poverty is not well understood. Our research aims is to build on and improve existing evidence examining whether aspects of occupant behaviour, beyond the use of heating systems, influence movements into and out of fuel poverty among households living in disadvantaged communities. Analyses are based on a large sample of households from some of the most deprived communities in Scotland UK, where official rates of fuel poverty are high, household circumstances and residential patterns fluctuate, and where interventions to address fuel poverty are concentrated. Using a longitudinal study design we are able to explore the impact of multiple aspects of occupant behaviours and behavioural changes on movements into and out of self-reported fuel poverty. This has not been done before and provides a more nuanced insight then has been previously possible.

## 3 Methods

### 3.1 Study context

Our study takes place in Glasgow, Scotland. Official fuel poverty in Scotland has stood at around a third or more of households since 2009 (Scottish Government, 2016a), despite over 100,000 energy efficiency measures being installed per annum and the number of dwellings achieving an Energy Performance Certificate (EPC) rating of C or above increasing by three-quarters from 2010 to 2015 (Scottish Government, 2017a). A new fuel poverty strategy and legislation recently introduced by the Scottish Government includes a statutory target to reduce fuel poverty to 15% of households by 2030, and 5% by 2040 via action on several fronts: continued investment in energy efficiency measures in social housing and for fuel poor households, alongside mandated improvements in private rented housing; extending access to affordable energy through a supply switching service for low income households, and the establishment of a public energy company; action to maximise incomes such as extending eligibility to the Winter Fuel and Cold Weather Payments subsidy schemes^3^; and partnering with third sector organisations to provide advice on heating use and control and energy saving behaviours (Scottish Government, 2018a). However, the regulation of the energy market, including price controls and financial support for renewable energy production, remain reserved powers (Scottish Government, 2017b).

Half of Glasgow's communities are classified as among the most deprived in Scotland (Scottish Government, 2016b); our study takes place in fifteen such communities. Fuel poverty in Glasgow stands at 34% of households, with 52% of dwellings achieving an EPC rating of C or above (Glasgow City Council, 2016). Incomes in Glasgow are low, with 17% of households living on a net income of less than £10,000 (US$13,500) and a third of all children living in poverty, and health is poor, with life expectancy for men 3.7 years lower than the national average (Understanding Glasgow, 2018). The context is a city with a relatively large social rented housing sector (36% of dwellings) where housing-led regeneration has been underway since the transfer of the public housing stock to a third sector landlord in 2003 (Gibb, 2003). Investment of £1.2 billion in improvements to the social housing stock over the following decade has comprised new heating systems, fabric insulation and re-roofings.

### 3.2 Data

The analysis draws upon four waves of household survey data collected in fifteen study communities in 2006, 2008, 2011 and 2015. In six of the smaller study areas undergoing extensive renewal, all occupied dwellings were selected for the survey and in the other nine random samples of addresses were selected in the first two surveys, with those properties where interviews had been conducted being re-contacted in the third and fourth surveys.^4^ The survey design is therefore a repeat cross-sectional survey with a nested longitudinal cohort. The surveys achieved samples of 5956 (response rate 50.3%), 4517 (47.5%), 3949 (45.4%) and 3471 (47.0%); these are considered respectable given declining response rates to surveys in recent years (Scottish Government, 2010) and lower response rates in Glasgow than in other districts (Scottish Government, 2013).

Characteristics of survey respondents are presented in Table 1. Compared with the adult population of the city as a whole, our sample from deprived areas is more likely to have a long-term illness (45% v 22%, limiting) and less likely to be in employment (22% v 47%). In household terms, our sample comprises more older person households (31% v 21%) and fewer adult-only households (43% v 60%), and is more likely to live in houses (35% v 27%) and in high-rise flats (33% v 7%).^5^ The current analysis uses the nested longitudinal sample, identified by matching names, addresses and household characteristics at each pair of consecutive survey waves, giving a total sample of 3297 longitudinal respondents with complete data at two time points (denoted T1 and T2). The use of longitudinal data is a major strength of this survey as it allows us to consider behaviours that impact on households' movements into (i.e. no fuel poverty at T1 but fuel poverty at T2) and out of (i.e. fuel poverty at T1 but not at T2) fuel poverty. Characteristics of this analytical sample are also presented in Table 1. It was broadly similar to the full survey although there was some suggestion that those included in the current analyses were more likely to report having a long-standing illness.

**Table 1:** Characteristics of full survey and analytical sample at T2.

	Full survey % (N = 8705)	Analytical sample % (N = 3297)
Sex		
Male	37.4	38.0
Female	62.6	62.0
Age		
16-24	2.6	1.7
25-39	19.6	19.4
40-54	28.9	28.2
55-64	18.1	17.1
65+	30.9	33.6
Household type		
Adult(s)	42.8	40.4
Single parent family	14.1	14.3
Two-parent family	11.8	11.8
Older person(s)	31.4	33.5
Household illness		
No LSI	54.4	48.0
Respondent has LSI	38.4	43.9
Other household member has LSI	7.2	8.1
House type		
Multi-storey flat	32.9	35.2
Other flat	31.6	32.5
House	35.5	32.3
Employment status		
Not working	78.3	80.0
Working full or part time	21.7	20.0
Vehicle ownership		
No access to car or van	74.5	74.8
Access to car or van	25.5	25.2

### 3.3 Dependent variable

The survey used an experiential (subjective) measure of fuel poverty. Respondents were asked how often they had difficulty meeting the cost of fuel bills, with response categories: never; occasionally; quite often; or very often. Three percent of respondents either did not know or considered the question not applicable (e.g. if they were not responsible for paying for energy), and are excluded from the analysis. For this analysis, we combine the response categories into a dichotomous measure of reported fuel poverty (any frequency of difficulty) versus not (never) at each wave and examine the determinants of changes in status on this measure between T1 and T2. (Table 2).

**Table 2:** Changes in fuel poverty during each time period and for entire longitudinal sample.^a^

	Wave 1-2 2006–2008	Wave 2-3 2008–2011	Wave 3-4 2011–2014	Total Sample
T1 to T2 change in fuel poverty (%)				
No fuel poverty at either time	56.2	55.3	58.8	56.6
Lose fuel poverty at T2	16.8	17.3	17.7	17.3
Gain fuel poverty at T2	15.9	17.9	13.1	15.8
Fuel poverty at both times	11.1	9.5	10.5	10.3
n	(1052)	(1241)	(1004)	(3297)
Fuel poverty gain in those with none at T1 (%)				
No fuel poverty at T2	78.0	75.6	81.8	78.2
Fuel poverty at T2	22.0	24.5	18.2	21.8
n	(758)	(908)	(721)	(2387)
Fuel poverty loss in those with fuel poverty at T1 (%)				
Fuel poverty at T2	39.8	35.4	37.1	37.4
No fuel poverty at T2	60.2	64.6	62.9	62.6
n	(294)	(333)	(283)	(910)

a Numbers for fuel poverty gain or loss are based on respondents with complete data for all potential determinants; numbers for change in fuel poverty are based on respondents with complete data for all potential confounding variables used in regression analyses.

### 3.4 Independent variables

Household characteristics and behaviours were grouped into four domains, as presented in Table 3, covering all but one of the domains presented in Fig. 1.^6^ Behavioural variables focussed on either most recent (T2) circumstances or changes in circumstances from T1 to T2, exploiting the longitudinal nature of the data. All variables were treated as categorical with the reference category (denoted [ref]) determined on the basis of ease of interpretation and/or maximising the number of participants to maintain statistical power. The first domain, household characteristics, included most recent household type, change in household size, and change in members of the household with long standing illness. The second domain, housing, comprised most recent dwelling type and home improvements or house moves reported in the 2–4 year period (dependent on the survey interval) immediately prior to T2. The third domain, employment and finances, covered changes in employment status, any reported difficulties in meeting the costs of food, repairs, rent, or council tax at T2, and changes in the number of such difficulties from T1 to T2. The final domain, social behaviours, focussed on T2, considering the number of amenities used in the past seven days, frequency of contact with family and friends, and the number of days on which respondent reported walking in the neighbourhood for 20 min or more.

**Table 3:** Most recent/changes in household, housing, employment & finances, and social behaviours.

	Definition	% among respondents without fuel poverty at T1 (N = 2387)	% among respondents with fuel poverty at T1 (N = 910)
Domain – Household			
Household type (T2)	Adult(s) [ref]	38.3	46.0
	Single parent family	12.9	18.0
	Two-parent family	11.2	13.4
	Older person(s)	37.7	22.5
Household Size (T1 to T2)	No change [ref]	78.2	78.8
	Decrease in numbers	11.8	11.9
	Increase in numbers	10.0	9.3
Household Illness (T1 to T2)	No LSI at T1 or T2 [ref]	39.5	36.0
	LSI at T1 and T2	32.6	31.9
	LSI gained by T2	18.1	23.5
	LSI lost by T2	9.8	8.6
Domain – Housing			
Dwelling type (T2)	Multi-storey flat [ref]	34.1	38.0
	Other flat	31.5	35.1
	House	34.4	26.9
Home improvements (pre-T2)	No [ref]	52.6	55.5
	Yes	47.4	44.5
House move (pre-T2)	No [ref]	90.7	87.7
	Yes	9.3	12.3
Domain – Employment and Finances			
Employment (T1 to T2)	Not working at T1 or T2 [ref]	73.3	77.9
	Gained work by T2	5.3	6.9
	Lost work by T2	5.6	5.1
	Working at T1 and T2	15.8	10.1
Financial Difficulties (T1 to T2)	No difficulties at T1 or T2 [ref]	67.8	11.4
	Gain/increase by T2	22.7	20.0
	Loss/decrease by T2	9.6	68.6
Difficulty paying for food (T2)	No [ref]	85.8	77.5
	Yes	14.2	22.5
Difficulty paying for repairs (T2)	No [ref]	93.5	89.8
	Yes	6.5	10.2
Difficulty paying rent (T2)	No [ref]	90.2	83.8
	Yes	9.7	16.2
Difficulty paying council tax (T2)	No [ref]	84.6	75.6
	Yes	15.4	24.4
Domain – Social Behaviours			
Amenities used (T2)^a^	None	6.8	6.5
	1 or 2	25.8	29.2
	3 or 4 [ref]	43.4	44.6
	5 or more	24.1	19.7
Contact with family (T2)	Most days [ref]	28.9	25.9
	Weekly	36.7	36.0
	Monthly	14.1	14.3
	Less often/never	20.2	23.7
Contact with friends (T2)	Most days [ref]	27.3	23.3
	Weekly	43.2	43.7
	Monthly	14.3	16.8
	Less often/never	15.2	16.2
Walk in the neighbourhood (T2)	5–7 days per week [ref]	34.1	33.7
	1–4 days per week	29.1	29.9
	Never	36.8	36.4

a From ten amenities: sports facility, social venue, park, post office, grocers, supermarket, shopping centre, library, community centre, place of worship.

### 3.5 Analysis

Behavioural factors associated longitudinally with households' movements into and out of fuel poverty were identified from logistic regression analyses based on 2387 respondents who were not in fuel poverty at T1 and 910 respondents who were in fuel poverty at T1 respectively. In the former case, regression models calculated the odds of moving into fuel poverty at T2 and, in the latter, the odds of moving out of fuel poverty by T2. Associations of movement into and out of fuel poverty were initially explored separately for each behaviour of interest using univariable models. Subsequent analyses used multivariable models to explore the impact of behaviours reciprocally adjusted for the impact of others included in the model. These models were developed using a two-stage process: (i) first, including all behavioural variables within a single domain that were strongly associated with fuel poverty in the preliminary analyses and (ii) including all behavioural variables, regardless of the domain, that were strongly associated with fuel poverty in the previous stage. Selection of behavioural factors with strong associations with fuel poverty was based on a p value for heterogeneity in odds ratios across the categories of <0.05. Odds ratios and confidence intervals are presented in the Results with confidence intervals based on robust standard errors to allow for clustering within individuals contributing to more than one time period.

### 3.6 Sensitivity analyses

In addition to these main analyses we also carried out a number of sensitivity analyses. Firstly, results presented here are based on the full longitudinal sample. However, as the data collection waves covered periods pre-, during and post-the 2008–12 recession we also repeated our analyses separately for each survey interval (waves 1–2, waves 2–3, waves 3–4) to explore whether the recession had an impact on our results. The results from these analyses did not differ markedly from those presented here. Secondly, the final analyses were based on respondents with complete data for all behavioural variables to allow direct comparison between them. However, we repeated the univariable analyses using all available data in each case and these results were almost identical to those presented here. Thirdly, our measure of fuel poverty was based on any mention of difficulty, but the original question asked for frequency. The number of respondents reporting frequent fuel poverty was rather smaller than those reporting any and so statistical power was limited. Nonetheless, results based on this more extreme measure were similar to those presented here. Finally, in order to better understand the role of illness in determining movement into or out of fuel poverty, additional analyses were performed based on specific long-standing illnesses experienced over the past year by the respondent: respiratory conditions (e.g. asthma, bronchitis), circulatory conditions (e.g. heart problems, high blood pressure) and mental health problems (stress anxiety or depression). The impact of each illness was examined in logistic regression models for moving into and out of fuel poverty, controlling for household type, change in household members, and, in multivariable models, adjusting for the other two health conditions. The results of these supplementary health analyses are reported below.

### 3.7 Limitations

We did not explore the influence of one of the behavioural domains, heating. While our focus was not on heating behaviours, it may be the case that including heating factors may alter some of the other relationships examined. The response rates to our survey are at or below 50%, with a likelihood the survey did not capture some of the most vulnerable households living in deprived areas, thus we may underestimate the impacts of some behavioural factors. Due to small numbers with finer temporal granularity, our analysis only covers change between two time points; looking at several time points would ensure that circumstances recorded at a point in time are not unusual or short-lived. Although our study is longitudinal and provides stronger indications of relationships, we cannot be certain about causality without knowing the precise timing of changes in circumstances during the survey intervals.

## 4 Results and discussion

### 4.1 Rates of fuel poverty

The rate of fuel poverty on our measure changed little over the first three survey waves (28.2% in Wave 1, 26.7% in Wave 2, 27.8% in Wave 3) and then fell to 23.5% in Wave 4. These rates of reported difficulty paying fuel bills have moved apart from official fuel poverty rates over time. Thus, in 2006, the experiential fuel poverty rate in our sample was 3 percentage points higher than the official fuel poverty rate of 24.7%, in 2008 the two were the same, but then the experiential rate dropped below the official rate of 32.9% by five points, and was 7 points lower than the official rate of 30.7% in 2015 (Scottish Government, 2016a, Table 30). It seems that whilst the official fuel poverty rate is said to have ‘broadly mirrored the growth in the fuel price index’ (Scottish Government, 2016a, p.64), this is not true of the experiential fuel poverty rate which is affected by many other factors, including occupant behaviours.

Table 2 presents moves in and out of fuel poverty in the longitudinal sample. The majority of households had a stable position over time, with approximately 56% reporting no difficulty paying for fuel at either time point in each longitudinal sample, and around 10% reporting difficulties at both time points. The remaining third of households divide into those who left fuel poverty over time (approximately 18%) and those who entered fuel poverty (16%). Where households did not report fuel poverty at the first time point, around a fifth (18–24%) moved into fuel poverty, i.e. reported difficulties paying for fuel, at the next. Where households reported fuel poverty at the first time point, around three-fifths (60–65%) moved out of fuel poverty, i.e. did not report difficulties paying for fuel at the next. Thus, there is more stability over time among those without experiential fuel poverty than there is among those with such fuel poverty at any time point.

### 4.2 Movements into fuel poverty

Analyses of movement into fuel poverty were based on respondents who did not report any fuel poverty at T1. Behavioural characteristics of these respondents are presented in Table 3, while Table 4 contains the results from the regression model for movements into fuel poverty over time. These results suggest that household structure has more impact than changing household size. Single parents are most likely to move into fuel poverty (Odds Ratio [OR] 2.27), possibly reflecting their vulnerable single source of low-income. The proportion of households comprising single parent families is three times higher in the most deprived areas (20%) than it is in the least deprived (6%), reflecting greater instability in household arrangements (NRS, 2015). Older person households were the least likely to move into fuel poverty (OR 0.69), perhaps because they are more conservative in their use of energy, receive winter fuel payments, and are not as badly affected as other households by changes in earnings and benefits following the recession and austerity measures (Whittaker, 2015). Lower rates of fuel poverty have been reported previously for people over 60 (Ahmad, 2013), retired households (BelaΪd, 2018) or retired couples (Legendre and Ricci, 2015). Bramley et al. (2017) found much higher rates of fuel poverty for older households, but only under the Boardman definition and much less so under other definitions. Changes in number of household members was not found to be associated with movements into fuel poverty.

**Table 4:** Impact of household, housing, employment and finances, and social behaviours on movement into fuel poverty: Odds ratios (95% CIs).

	Unadjusted	Adjusted within Domain	Adjusted Across Domains
*Domain – Household:*			
Household type (T2)			
Adult(s)	1.00	1.00	1.00
Single parent family	1.94 (1.47, 2.56)	1.98 (1.49, 2.64)	2.27 (1.52, 3.39)
Two-parent family	1.05 (0.76, 1.43)	1.09 (0.80, 1.51)	1.05 (0.68, 1.62)
Older person(s)	0.42 (0.32, 0.54)	0.40 (0.31, 0.52)	0.69 (0.48, 0.99)
*p*	*<0.001*	*<0.001*	*<0.001*
Household Size			
No change	1.00		
Decrease in numbers	1.04 (0.77, 1.41)		
Increase in numbers	0.98 (0.70, 1.35)		
*p*	*0.95*		
Household Illness			
No LSI at T1 or T2	1.00	1.00	1.00
LSI at T1 and T2	0.83 (0.65, 1.05)	1.20 (0.93, 1.54)	0.87 (0.60, 1.25)
LSI gained by T2	1.26 (0.97, 1.64)	1.56 (1.19, 2.05)	1.06 (0.72, 1.56)
LSI lost by T2	1.05 (0.74, 1.48)	1.33 (0.92, 1.91)	0.86 (0.53, 1.41)
*p*	*0.03*	*0.02*	*0.75*
*Domain – Housing:*			
Dwelling type (T2)			
Multi-storey flat	1.00	1.00	1.00
Other flat	0.66 (0.52, 0.84)	0.66 (0.52, 0.84)	0.82 (0.58, 1.16)
House	0.56 (0.45, 0.72)	0.56 (0.45, 0.72)	0.81 (0.57, 1.15)
*p*	*<0.001*	*<0.001*	*0.39*
Home improvements (pre-T2)			
No	1.00		
Yes	1.11 (0.91, 1.35)		
*p*	*0.29*		
House move (pre-T2)			
No	1.00		
Yes	1.27 (0.92, 1.75)		
*p*	*0.14*		
*Domain – Employment and Finances:*			
Employment			
Not working at T1 or T2	1.00	1.00	1.00
Gained work by T2	0.96 (0.62, 1.48)	0.68 (0.36, 1.30)	0.53 (0.29, 0.99)
Lost work by T2	1.11 (0.73, 1.67)	0.84 (0.45, 1.55)	0.76 (0.40, 1.43)
Working at T1 and T2	0.64 (0.48, 0.87)	0.48 (0.31, 0.73)	0.42 (0.27, 0.68)
*p*	*0.03*	*0.005*	*<0.001*
Financial Difficulties			
No difficulties at T1 or T2	1.00	1.00	1.00
Gain/increase by T2	31.63 (24.22, 41.30)	3.85 (2.33, 6.36)	3.25 (1.93, 5.48)
Loss/decrease by T2	2.91 (1.96, 4.34)	2.34 (1.55, 3.52)	2.11 (1.40, 3.18)
*p*	*<0.001*	*<0.001*	*<0.001*
Difficulty paying for food (T2)			
No	1.00	1.00	1.00
Yes	45.46 (32.69, 63.23)	9.69 (6.18, 15.20)	9.72 (6.09, 15.49)
*p*	*<0.001*	*<0.001*	*<0.001*
Difficulty paying for repairs (T2)			
No	1.00	1.00	
Yes	11.24 (7.80, 16.21)	1.51 (0.90, 2.53)	
*p*	*<0.001*	*0.12*	
Difficulty paying rent (T2)			
No	1.00	1.00	1.00
Yes	13.45 (9.81, 18.42)	2.16 (1.35, 3.46)	2.28 (1.41, 3.70)
*p*	*<0.001*	*<0.001*	*<0.001*
Difficulty paying council tax (T2)			
No	1.00	1.00	1.00
Yes	19.35 (14.84, 25.22)	3.00 (1.94, 4.65)	3.29 (2.09, 5.16)
*p*	*<0.001*	*<0.001*	*<0.001*
*Domain – Social Behaviours*			
Amenities used (T2)			
None	0.85 (0.56, 1.29)		
1 or 2	0.86 (0.67, 1.10)		
3 or 4	1.00		
5 or more	1.05 (0.82, 1.34)		
*p*	*0.44*		
Contact with family (T2)			
Most days	1.00	1.00	1.00
Weekly	0.91 (0.71, 1.18)	0.93 (0.72, 1.20)	0.99 (0.71, 1.38)
Monthly	1.25 (0.91, 1.71)	1.30 (0.95, 1.78)	1.23 (0.80, 1.90)
Less often/never	1.61 (1.23, 2.12)	1.65 (1.25, 2.18)	1.80 (1.21, 2.67)
*p*	*<0.001*	*<0.001*	*0.01*
Contact with friends (T2)			
Most days	1.00		
Weekly	0.93 (0.73, 1.18)		
Monthly	1.10 (0.80, 1.51)		
Less often/never	1.24 (0.91, 1.68)		
	0.22		
Walk in the neighbourhood (T2)			
5–7 days per week	1.00	1.00	1.00
1–4 days per week	0.74 (0.58, 0.95)	0.72 (0.56, 0.93)	0.77 (0.55, 1.08)
Never	0.80 (0.64, 1.01)	0.78 (0.62, 0.99)	0.91 (0.64, 1.29)
*p*	*0.04*	*0.02*	*0.32*

Households where a member(s) acquires a long-standing illness or disability have a higher likelihood of moving into fuel poverty than others (OR 1.56), although the effect is attenuated once other domains are included. This may partly reflect a shift in behaviours including being at home more, additional energy use and other additional costs, in accord with earlier suggestions that fuel costs would be higher due to a number of health conditions (Hodges et al., 2016). Consistent with our finding, Bramley et al. (2017) also estimated the rate of fuel poverty in Scotland to be higher among those with a long-term illness or disability. In addition, problems of low-incomes and cuts to disability benefits since 2010 are well documented, with one estimate suggesting that the disabled bear three times as much of the burden of austerity measures as their numbers would justify (Duffy, 2014). Issues of high mortality and morbidity in the Glasgow region are well known and often attributed to either cultural factors or the legacy of the city's industrial past (Walsh et al., 2010). In places with concentrated, enduring unemployment people are said to perceive their health conditions as more limiting, thus constraining themselves to worklessness and low income, which may exacerbate fuel poverty (Webster, 2013). Supplementary analysis suggested that suffering mental health issues, at either or both time points, more than doubled the odds that the respondent would move into fuel poverty over time (OR 2.27–2.74). This adds new evidence of a different kind to earlier findings that improvements in mental health often follow interventions to tackle fuel poverty (Allen, 2005; Green and Gilberston, 2008; Howden-Chapman et al., 2007), due either to reductions in perceived financial strain or increased value for money and sense of control (Liddell and Morris, 2010).

Within the housing domain, dwelling type had a strong effect. People living in multi-storey flats were more likely to enter fuel poverty than those living in other types of flats (OR 0.66) or houses (OR 0.56). However, this was attenuated after adjustment for other domains. While earlier research in England reported higher rates of fuel poverty in houses, recent analysis for Scotland shows higher fuel poverty for those living in flats, on most measures of fuel poverty (Ahmad, 2013; Bramley et al., 2017). Although multi-storey buildings have higher energy efficiency than low-rise dwellings, they also contain more expensive forms of electrical space heating, which occupants struggle to use economically or effectively (Scottish Government, 2016a, Tables 5 and 6, Figure 13). Neither moving home, nor having home improvement works done, had strong effects upon the chances of moving into fuel poverty.

The results show the protective function of employment. Those who remained out of work over time were twice as likely to move into fuel poverty as those who gained work (OR 0.53) or remained in employment (OR 0.42). The increased likelihood of being fuel poor if unemployed has been reported previously (BelaΪd, 2018; Ahmad, 2013). Employment seeking behaviours are therefore important, although in our study only one-in-seven (14%) of working-age adults out of work sought employment over time, again indicating the potentially limiting nature of participants' behaviour. Fuel poverty was also associated with poverty in other domains, with the likelihood of moving into fuel poverty at least doubled if respondents experienced other financial difficulties at any point, and trebled if those difficulties increased over time (OR 3.25). Budgeting problems were also relevant, with the likelihood of entering fuel poverty at least doubling where there were difficulties paying other housing-related costs such as rent (OR 2.28) or council tax (OR 3.29). However, where a household experienced difficulties paying for food, the odds of reporting fuel poverty were increased nearly ten-fold (OR 9.72) indicating the close association between paying for food and energy, two essential budget items where households exercise discretion, often cut-back, and make trade-offs (Citizens Advice Scotland, 2016).

In the social behaviours domain, no marked associations were evident with either the use of public amenities or the frequency of contact with friends. However, those who had contact with wider family members most days were nearly half as likely to move into fuel poverty as those who rarely or never saw their relatives (OR 1.80), after controlling for the effects of the other domains. Contact with wider family members may offer several benefits including spending time out of the home and/or at the other family member's home, getting advice about budgeting and finances, and receiving financial assistance to help pay bills from time to time. Those who walked in their neighbourhood most days of the week were more likely to move into fuel poverty than those who never walked locally (OR 0.78). The fact that this effect was attenuated by other domains such as employment and finances suggests that regular local walking may be more of a response to fuel poverty than a protective behaviour.

### 4.3 Movements out of fuel poverty

Table 3, Table 5 show the equivalent characteristic and regression model for movements out of fuel poverty among respondents who reported being in fuel poverty at T1. In the household domain, single parenthood and the presence (or absence) of long-standing illness did not affect movements out of fuel poverty. However, older person households were more likely to move out of fuel poverty (OR 1.48) after adjustment across all domains, mirroring the result in the previous regression. Results from supplementary analysis again indicate that having a mental health problem at either time point halved the chances that someone would move out of fuel poverty (OR 0.44–0.50). Conversely, having a circulatory condition at both time points increased the likelihood of moving out of fuel poverty (OR 2.50), possibly reflecting the role of additional supplementary benefit income available to adults in such circumstances.

**Table 5:** Impact of household, housing, employment and finances, and social behaviours on movement out of fuel poverty: Odds ratios (95% CIs).

	Unadjusted	Adjusted within Domain	Adjusted Across Domains
*Domain – Household:*			
Household type (T2)			
Adult(s)	1.00	1.00	1.00
Single parent family	0.86 (0.59, 1.24)	0.86 (0.59, 1.24)	0.74 (0.43, 1.27)
Two-parent family	0.95 (0.63, 1.41)	0.95 (0.63, 1.41)	0.97 (0.54, 1.76)
Older person(s)	2.81 (1.92, 4.13)	2.81 (1.92, 4.13)	1.48 (0.92, 2.37)
*p*	*<0.001*	*<0.001*	*<0.001*
Household Size			
No change	1.00		
Decrease in numbers	1.29 (0.84, 1.09)		
Increase in numbers	1.08 (0.68, 1.71)		
*p*	*0.50*		
Household Illness			
No LSI at T1 or T2	1.00		
LSI at T1 and T2	0.97 (0.70, 1.34)		
LSI gained by T2	1.09 (0.77, 1.55)		
LSI lost by T2	0.96 (0.58, 1.59)		
*p*	*0.93*		
*Domain – Housing:*			
Dwelling type (T2)			
Multi-storey flat	1.00	1.00	1.00
Other flat	0.76 (0.56, 1.04)	0.78 (0.57, 1.07)	0.58 (0.39, 0.97)
House	1.54 (1.08, 2.18)	1.51 (1.06, 2.14)	1.33 (0.81, 2.20)
*p*	*<0.001*	*0.001*	*0.001*
Home improvements (pre-T2)			
No	1.00		
Yes	1.07 (0.81, 1.40)		
*p*	*0.65*		
House move (pre-T2)			
No	1.00	1.00	
Yes	0.60 (0.40, 0.89)	0.66 (0.44, 0.98)	
*p*	*0.01*	*0.04*^a^	
*Domain – Employment and Finances:*			
Employment			
Not working at T1 or T2	1.00		
Gained work by T2	0.92 (0.54, 1.55)		
Lost work by T2	1.03 (0.55, 1.91)		
Working at T1 and T2	1.19 (0.76, 1.86)		
*p*	*0.87*		
Financial Difficulties			
No difficulties at T1 or T2	1.00	1.00	
Gain/increase by T2	0.04 (0.02, 0.07)	0.85 (0.36, 2.02)	
Loss/decrease by T2	0.47 (0.26, 0.86)	1.06 (0.57, 1.97)	
*p*	*<0.001*	*0.70*	
Difficulty paying for food (T2)			
No	1.00	1.00	1.00
Yes	0.03 (0.02, 0.006)	0.06 (0.03, 0.10)	0.05 (0.03, 0.09)
*p*	*<0.001*	*<0.001*	*<0.001*
Difficulty paying for repairs (T2)			
No	1.00	1.00	1.00
Yes	0.08 (0.05, 0.15)	0.41 (0.18, 0.91)	0.44 (0.20, 0.98)
*p*	*<0.001*	*0.03*	*0.05*
Difficulty paying rent (T2)			
No	1.00	1.00	1.00
Yes	0.10 (0.06, 0.16)	0.28 (0.15, 0.52)	0.28 (0.15, 0.54)
*p*	*<0.001*	*<0.001*	*<0.001*
Difficulty paying council tax (T2)			
No	1.00	1.00	1.00
Yes	0.07 (0.04, 0.10)	0.23 (0.14, 0.38)	0.23 (0.13, 0.39)
*p*	*<0.001*	*<0.001*	*<0.001*
*Domain – Social Behaviours*			
Amenities used (T2)			
None	1.82 (0.97, 3.43)		
1 or 2	0.83 (0.60, 1.14)		
3 or 4	1.00		
5 or more	0.86 (0.60, 1.24)		
*p*	*0.10*		
Contact with family (T2)			
Most days	1.00	1.00	1.00
Weekly	0.90 (0.63, 1.28)	0.91 (0.64, 1.29)	0.93 (0.58, 1.51)
Monthly	0.93 (0.60, 1.46)	0.94 (0.59, 1.47)	0.98 (0.50, 1.90)
Less often/never	0.56 (0.39, 0.82)	0.55 (0.38, 0.81)	0.70 (0.41, 1.20)
*p*	*0.01*	*0.01*	*0.57*
Contact with friends (T2)			
Most days	1.00	1.00	
Weekly	1.03 (0.72, 1.47)	1.00 (0.68, 1.46)	
Monthly	0.64 (0.42, 0.97)	0.67 (0.43, 1.04)	
Less often/never	0.78 (0.50, 1.20)	0.81 (0.50, 1,30)	
*p*	*0.06*	*0.18*	
Walk in the neighbourhood (T2)			
5–7 days per week	1.00	1.00	1.00
1–4 days per week	1.12 (0.80, 1.57)	1.15 (0.82, 1.61)	0.92 (0.57, 1.48)
Never	1.46 (1.05, 2.02)	1.51 (1.09, 2.09)	1.53 (0.97, 2.42)
*p*	*0.07*	*0.04*	*0.06*

a House move is not included in the final model as it was no longer associated with losing fuel poverty after adjustment for all other variables.

In the housing domain, movement out of fuel poverty was less likely for those living in ‘other’ flats (OR 0.58). Home improvement works had no effect on the likelihood of moving out of fuel poverty. Previous research has reported a lower likelihood of fuel poverty among those whose home has been refurbished, using the LIHC indicator (BelaΪd, 2018). Our contrary results using an experiential measure of fuel poverty raises questions about whether such works are targeted or suitable for those in need (Thomson et al., 2013), and whether post-improvement advice and support is (un)available to assist users. Our finding may, however, be consistent with other research on warmth interventions which found the rebound effect, or taking of extra comfort heating after improvements, to be greater in lower income areas where rates of fuel poverty were higher (Webber et al., 2015). Moving home lowered the likelihood of moving out of fuel poverty, which could be a result of the costs of moving itself, or a product of moving into improved homes with different heating systems to those the occupants are familiar with. However, the impact of moving home was attenuated by adjustment for other domains, suggesting that house moves may be related to other factors such as household and employment change.

Changes in employment status had no effect on movements out of fuel poverty, providing new evidence to add to that of the effects of current employment status (BelaΪd, 2018; Ahmad, 2013). This may be due to the predominance of low-wage, temporary and part-time employment in the post-recession era, particularly for more disadvantaged groups. The odds of moving out of fuel poverty were more than halved among those having difficulties paying housing-related costs such as repairs (OR 0.44), rent (OR 0.28) or council tax (OR 0.23). The close relationship between fuel poverty and food insecurity (Tuttle and Beatty, 2017) is evident again, with people experiencing difficulties paying for food having very low probability of moving out of fuel poverty (OR 0.05).

Those who rarely or never saw their relatives had much lower odds of moving out of fuel poverty (OR 0.55); the attenuation of this association after control for other domains suggests that family support may be beneficial financially and/or emotionally (with the maintenance of relationships). Those who never walked around their neighbourhood had a higher likelihood of moving out of fuel poverty (OR 1.51), which in a context of relatively low levels of physical activity in disadvantaged areas in Scotland (McLean et al., 2017, Figure 3C) may indicate that where walking is regularly undertaken, it may be partly a product of poverty and poor home circumstances that encourages people to spend time outdoors.

## 5 Conclusions and policy implications

We took a different approach to the investigation of fuel poverty to past studies. First, we conceptualised occupant behaviour to embrace a wider range of individual and household characteristics as influential upon attitudes to energy conservation, use of energy, and ability to manage energy bills, thereby affecting fuel poverty. Second, we used an experiential measure of fuel poverty as an alternative to a technical measure based on modelled household incomes and energy requirements. Third, we used longitudinal survey data to examine movements into and out of fuel poverty, something not done previously. In doing so, we included a wider range of behavioural variables relating to household and employment dynamics, health, household budgets, and social relations. Thus, our study is unique in several respects enabling us to present new evidence.

Our new findings include the fact that single parent households were more likely to move into fuel poverty, and older person households less likely. The onset of long-term health conditions was also more likely to be followed by fuel poverty, and we highlighted for the first time the effect of poor mental health on movements into fuel poverty, rather than merely being a consequence of it. Our results also showed a close connection between fuel poverty and food insecurity as an influence upon movements both into and out of fuel poverty. The role of social connectivity is foregrounded in our findings, with wider family relations found to be both protective and curative of fuel poverty. In relation to movements out of fuel poverty, we reported a lack of effect of home improvements and limited impacts from gaining employment, at least for residents of deprived communities in the current era. Additionally, our findings showed that moving home, which often takes the form of relocation during regeneration programmes, reduced the chances of movement out of fuel poverty, again raising questions about the effectiveness of interventions.

The policy implications of these findings have to be considered against the backdrop of the situation in Scotland, where rates of fuel poverty have been high (a quarter to a third of households) for the last fifteen years or more, despite the delivery of large-scale energy efficiency programmes to residential homes over the same period (Scottish Government, 2016a). First, we argue that fuel poverty should be conceived slightly differently, as problem definition affects policy agendas (Knill and Tosun, 2012). The term ‘fuel poverty’ may cause tunnel vision among policy-makers, suggesting a focus on issues pertaining to fuel costs and incomes, with energy efficiency interventions as the bridge between the two. We suggest that policy might contemplate an alternative approach whereby the problem is seen as one of ‘warmth & energy deprivation’, i.e. the lack of something that the majority have, which may be due to its unaffordability or unattainability. In this perspective, a lack of warmth and energy can be seen as an issue of both justice and capability (Walker and Day, 2012). While people need to have incomes, homes and heating/energy systems that make warmth and energy affordable (the fuel poverty view), the end state also needs to be attainable in a practical sense, i.e. the feasible combination of those three elements by the occupant. Furthermore, the individual has to be capable of attaining (suitably using space, heating and appliances) and affording (paying for) warmth and energy; it is not simply a technical/financial matter to do this. Thus, a broader policy understanding of fuel poverty would see human agency playing a part and occupant behaviour as an additional driver.

There is also a case for alternative measurement of the problem. Our experiential measure produced rates of fuel poverty below the official rate in recent years, supporting the argument that the official ratio measure does ‘not reflect well those in the underlying problems’ and that ‘it is unduly sensitive to changes in price levels as well as to technicalities within its calculation’ (Hills, 2012, p.2). Rather than rely upon a single indicator of fuel poverty (Tirado-Herrero, 2017), the evidence base for fuel poverty policy should be expanded through examination of the relationship(s) between technical and experiential measures. In addition, to support different policy interventions, more research is required on occupant behaviour in all aspects that may pertain to warmth and energy deprivation, such as how occupants respond to illness, to unemployment, and to acute or chronic insufficiency of household budget. Furthermore, longitudinal studies that examine pathways from occupant behaviour to fuel poverty via the interim outcomes of energy conservation, use of energy and management of energy bills (Fig. 1) are necessary. Insights may also be gained by examining movements out of fuel poverty, which were found to be more common than movements into fuel poverty in the current study, but are rarely investigated by a policy system focused on simple, descriptive performance indicators that cannot identify causal relationships (Lehtonen, 2015). The Scottish Government has shown recent interest in a stronger focus on low income and on the ‘lived experience’ of fuel poverty, which would correspond with the use of alternative measures of both fuel poverty and occupant behaviour (Scottish Government, 2018a).

Our findings possibly indicate that a higher post-improvement standard should be targeted by energy efficiency interventions: the mean post-improvement energy efficiency rating is 7.4 on the 0–10 National Home Energy Rating (NHER) scale (Wilson et al., 2012). Scottish Government has recently set a higher target of EPC grade B for social housing and fuel-poor households, but a lower standard of EPC C has been set for private sector housing (Scottish Government, 2018b). It may also be the case that more post-intervention support should be offered to occupants, after home improvements or relocation, for example to cope with new heating and energy systems or with other post-intervention costs. This has been identified as a missing element of many such programmes (Faulk, 2015). Scottish Government has at least acknowledged that ‘as well as improving the physical fabric of homes, it is equally important we support home owners to change their behaviours to that they can control their heating system … and get the most out of energy efficient improvements’ (Scottish Government, 2018a, p.19).

Lastly, one consequence of a stronger focus on occupant behaviour would be an expansion of the policy definition of vulnerability - *to* as well as *from* fuel poverty - including exploration of the feasibility of a qualitative risk-based metric (Mould and Baker, 2017a). Vulnerability should be taken to cover both physical and mental health, incorporating both direct effects as well as how people respond to health issues within the socio-economic context in which they reside, which would allow consideration of the moderating effects of social and economic factors upon health impacts (Dodds, 2016). Vulnerability as a cause and consequence of occupant behaviour is not only health-related but also social, including the nature of a person's social contacts and support, and their relationship status, particularly where these are volatile. Following a recommendation about local collaborations from its working group on fuel poverty, the Scottish Government recently supported partnership working between Home Energy Scotland and both NHS advice lines and the new Social Security Agency for Scotland to target help to vulnerable fuel poor households (Sigsworth, 2016; Scottish Government, 2018a). A wider range of partnerships, including between energy advice organisations and family and welfare support agencies, could be developed in order to better identify and support those at risk of, and experiencing, warmth and energy deprivation.

## Footnotes

1 National Energy Action UK reported fuel poverty rates of 12% in England, 30% in Wales, 35% in Scotland and 42% in Northern Ireland: *UK Fuel Poverty Monitor 2014–15*.

2 Warm Front was the UK Government's main programme for tackling fuel poverty in England from 2000 to 2013. For those in the private housing sector in receipt of certain state benefits, the scheme provided a mixture of heating system improvements, insulation works, and energy advice (Green and Gilbertson, 2008).

3 The Winter Fuel Payment is an additional lump sum paid to people over pensionable age to assist with energy costs in winter. The Cold Weather Payment is a sum paid to households in receipt of certain state benefits for every week that temperatures are below zero degrees Celsius for seven consecutive days.

4 Further details of the study design are given in Egan et al. (2010), and of the surveys in Mason and Kearns (2017).

5 Our study areas do not match official neighbourhoods and therefore population data are unavailable for them. Most of the differences noted above reflect the fact that the study is in some of the most deprived communities in the city. Sources for the city-wide data include: Scottish House Condition Survey 2011–13; NHS Greater Glasgow & Clyde Health and Wellbeing Survey 2014; and Scottish Household Survey 2015.

6 We do not include heating/energy use and thermal comfort, and combine household structure and dynamics with health and wellbeing for convenience in the statistical modelling.
